# *TGF-β1* and *TGF-βR1* variants are associated with clinical outcomes in smoking-related head and neck cancer patients treated with chemoradiation through modulating *microRNA*-mediated regulation

**DOI:** 10.1007/s00262-024-03672-y

**Published:** 2024-03-30

**Authors:** Zihao Niu, Peng Sun, Mark E. Zafereo, Hongliang Liu, Peng Wei, Jia Wu, Neil D. Gross, Sanjay Shete, Qingyi Wei, Guibin Zheng, Andy G. Sikora, George A. Calin, Guojun Li

**Affiliations:** 1 Department of Head and Neck Surgery, Unit 1445, The University of Texas MD Anderson Cancer Center, 1515 Holcombe Boulevard, Houston, TX 77030 USA; 2 Department of Otolaryngology Head and Neck Surgery, Beijing Tongren Hospital, Capital Medical University, Beijing, 100730 China; 3 Department of Otolaryngology, The First Affiliated Hospital of Soochow University, Suzhou, 215006 China; 4 Department of Medicine, Duke University School of Medicine, Durham, NC 27710 USA; 5 Department of Biostatistics, The University of Texas MD Anderson Cancer Center, Houston, TX 77030 USA; 6 Department of Imaging Physics, The University of Texas MD Anderson Cancer Center, Houston, TX 77030 USA; 7 Department of Epidemiology, The University of Texas MD Anderson Cancer Center, Houston, TX 77030 USA; 8 Department of Thyroid Surgery, the Affiliated Yantai Yuhuangding Hospital of Qingdao University, Yantai, 264000 Shandong China; 9 Department of Translational Molecular Pathology, The University of Texas MD Anderson Cancer Center, Houston, TX 77030 USA

**Keywords:** *TGF-β*-related genetic variants, Smoking status, microRNA binding site, Smoking-related head and neck cancer, Survival biomarkers

## Abstract

TGF-β1 and TGF-βR1 play important roles in immune and inflammatory responses. Genetic variants of *TGF-β1* rs1800470 and *TGF-βR1* rs334348 have emerged as potentially prognostic biomarkers for HPV-related head and neck cancer, while their prognostic effect on survival of smoking-related head and neck cancer remains unknown. This study included 1403 patients with smoking-related head and neck cancer, and all these patients were genotyped for *TGF-β1* rs1800470 and *TGF-βR1* rs334348. Both univariate and multivariate analyses were performed to evaluate associations between the two functional genetic variants in *microRNA* binding sites of *TGF-β1* and *TGF-βR1* and survivals. Patients with *TGF-β1* rs1800470 CT or CC genotype had 30–35% risk reductions for OS, DSS, and DFS compared to patients with TT genotype among overall patients, ever smokers, and patients administered chemoradiation. Furthermore, patients with *TGF-βR1* rs334348 GA or GG genotype had significant 50–60% risk reductions for OS, DSS, and DFS compared to patients with AA genotype among overall patients and patients administered chemoradiation; among ever smokers, the risk reductions even reached 60–70%. The TCGA dataset was used for validation. These findings suggest that *TGF-β1* rs1800470 and *TGF-βR1* rs334348 significantly affect survival outcomes in patients with smoking-related head and neck cancer, especially in the subgroups of ever smokers and patients treated with chemoradiation. These genetic variants may serve as prognostic indicators for patients with smoking-related head and neck cancer and could play a role in advancing the field of personalized chemoradiation, thereby improving patient survival and quality of life.

## Introduction

Head and neck squamous cell carcinoma (HNSCC) is the seventh most common cancer globally and has a 5-year survival rate of 40–50%, and its incidence is increasing [1]. In 2020, there were nearly 1,000,000 new cases of HNSCC and over 400,000 fatalities from HNSCC around the world, highlighting the considerable threat to public health posed by this disease [1]. Although tobacco and alcohol are the most important etiologic agents in HNSCC, recently, it has been well recognized that approximately 10–20% of HNSCC, chiefly oropharyngeal cancer, are associated with high-risk HPV infection [2].

The smoking-related head and neck cancer is a key subtype of HNSCC that originates in the oral cavity, hypopharynx, and larynx and is predominantly associated with smoking and alcohol consumption, which is markedly aggressive and often accompanied by nonspecific symptoms during the early stages, so patients are susceptible to misdiagnosis and delayed diagnosis [3, 4]. Despite continuous enhancements in recent years in treatments for smoking-related head and neck cancer, including surgery, chemotherapy, and radiotherapy, the prognosis for patients with smoking-related head and neck cancer has not significantly improved [5]. Notably, patients diagnosed with smoking-related head and neck cancer who have similar clinical and pathological characteristics and receive the same therapeutic interventions sometimes experience substantially different clinical outcomes [6–8].

The vital role of smoking in the etiology of smoking-related head and neck cancer may be attributed in part to the detrimental effects of chemical compounds found in tobacco smoke, including reactive oxygen species and nitrogen species, on macromolecules such as lipids, proteins, and nucleic acids; furthermore, the oxidative stress generated by the interaction of smoking-induced oxygen species and nitrogen species plays a significant part in promoting inflammation and cancer progression [9]. Previous research elucidated that inflammatory cytokines may play a pivotal role in the prognosis of patients with smoking-related head and neck cancer [10]. However, research on utilizing biomarkers to predict the prognosis of patients with smoking-related head and neck cancer may be limited.

The host’s immune response and the reaction to chronic inflammation are significant biological contributors to both the oncogenesis and prognosis of smoking-related head and neck cancer [11]. Transforming growth factor β1 (TGF-β1) exerts a notable impact on tumor development and the immune microenvironment by modulating various cellular processes, including differentiation, proliferation, migration, and apoptosis [12]. Transforming growth factor β receptor 1 (TGF-βR1), a single-pass serine/threonine kinase receptor, is a vital downstream component within the TGF-β signaling cascade, and absence of TGF-βR1 affects the function of this pathway [13].

Several studies [14–17] have shown that single nucleotide polymorphisms (SNPs) within miRNA binding sites, such as *TGF-β1* rs1800470 (merged from NCBI SNP rs1982073; T869C; codon 10 of exon 1; encoding Leu10Pro), which binds to miRNA-187, and *TGF-βR1* rs334348, which binds to miRNA-628-5p, may predict the clinical outcomes of patients with breast cancer and oropharyngeal SCC via their potential function to exert influence on gene expression regulation. *TGF-βR1* rs334348 is in strict linkage disequilibrium and is located in the 3’ untranslated region of *TGF-βR1* [14]. These SNPs at miRNA binding sites may subsequently induce alterations in cellular homeostasis, ultimately contributing to an individual’s predisposition to cancer risk and impacting disease prognosis [14, 18, 19]. Findings also suggest the potential involvement of these single nucleotide polymorphisms in the predisposition to and progression of specific diseases, underscoring the prospective clinical relevance of these genetic variants [20, 21]. Notably, our most recent study suggested that both *TGF-β1* rs1800470 and *TGF-βR1* rs334348 might significantly affect the survival of patients with human papillomavirus (HPV)-associated oropharyngeal cancer [22], while it remains unknown whether the same genetic variants of *TGF-β1* and *TGF-βR1* impact the clinical outcomes of smoking-related head and neck cancer. Since these two functionally significant genetic variants of *TGF-β1* and *TGF-βR1* operate within the same pathway, it may allow us to evaluate the comprehensive impact of *TGF-β1* rs1800470 and *TGF-βR1* rs334348 on survival of smoking-related head and neck cancer [23]. Assessing effects of *TGF-β1* rs1800470 and *TGF-βR1* rs334348 might reveal stronger associations between individual *TGF-β1* rs1800470 or *TGF-βR1* rs334348 variants and outcomes for patients with smoking-related head and neck cancer.

In the present study, we assessed the association of *TGF-β1* rs1800470 and *TGF-βR1* rs334348 with the prognosis of patients with smoking-related head and neck cancer overall and stratified by smoking status and treatment. The discovery and analysis of genetic variants that carry significant prognostic value could help advance the development of tailored therapeutic strategies.

## Materials and methods

### Study patients

In a comprehensive molecular epidemiology investigation of HNSCC carried out at The University of Texas MD Anderson Cancer Center from January 1998 to September 2012, a total of 1403 consecutive patients diagnosed with smoking-related head and neck cancer, including SCC of oral cavity, SCC of hypopharynx, and SCC of larynx, were enrolled. The study targeted newly diagnosed individuals with histologically verified, previously untreated smoking-related head and neck cancer, imposing no limitations on factors such as age, sex, race and ethnicity, cancer stage, or histological classification. This study was approved by the Institutional Review Board of MD Anderson Cancer Center and was performed in accordance with the Declaration of Helsinki, and all participants provided written informed consent prior to enrollment. Upon entering the study, patients were asked to complete an epidemiological questionnaire encompassing demographic information and risk factors, including smoking and alcohol use status. Furthermore, a 30mL pretreatment blood sample was procured from each patient for genotyping. Throughout the treatment period and subsequent follow-up, patients underwent routine clinical assessments and radiological examinations [18]. In-depth information pertaining to patient enrollment, follow-up, and the epidemiological and clinical data collected from the study patients has been previously reported [24].

### Genotyping of TGF-β1 rs1800470 and TGF-βR1 rs334348

Genomic DNA was extracted from leukocyte cell pellets with the QIAamp DNA Blood Mini Kit (QIAGEN Inc., Valencia, CA), following the manufacturer’s protocol. The genomic DNA was genotyped with the Illumina HumanOmniExpress-12v1 BeadChip as described in our previous paper [25]. For some patients with genotyping missing, genotyping was performed using the polymerase chain reaction–restriction fragment length polymorphism method as previously described [26]. Quality control measures were implemented, including repeated analysis of a randomly selected 10% of samples; results were 100% concordant with the initial results. For validation, the epidemiological, clinical, and survival data from the TCGA dataset and the genotyping of the 2 SNPs from the NIH dbGaP database were extracted on 312 smoking-related head and neck cancer patients, for whom such data was available for analysis.

### Statistical analysis

The primary endpoints of this investigation were overall survival (OS), disease-specific survival (DSS), and disease-free survival (DFS). OS was defined as the time from the first appointment at The University of Texas MD Anderson Cancer Center to death from any cause or date of the last follow-up. Participants who were alive at the end of the study period or lost to follow-up were considered censored. DSS was defined as the time from the first appointment to death from disease or the date of the last follow-up. Recurrent disease was defined as the appearance of a new lesion of the same histology verified by biopsy (incisional, excisional, or needle biopsy), the reappearance of any lesion that had disappeared, or the development of tumor-related symptoms. DFS was computed from the date of the end of treatment to the date of the last follow-up or the date of clinically detectable recurrent cancer (local, regional, or distant). Participants who were recurrence free or lost to follow-up were considered censored.

SAS software (version 9.4; SAS Institute, Cary, NC) was used for all statistical analyses. All statistical tests were two-sided, with *P* < 0.05 deemed indicative of statistical significance. In the univariate analysis, we evaluated the survival impact of epidemiological and clinical factors, such as age, sex, race and ethnicity, smoking status, alcohol use status, index tumor stage, comorbid conditions, and treatment. Some variables that did not exhibit statistically significant prognostic value in the univariate analysis were nonetheless incorporated into the main-effects and final multivariable models, given their relevance in epidemiological and clinical contexts. We applied the Kaplan-Meier method to compare survival rates among smoking-related head and neck cancer patients with distinct genotypes and calculated the log-rank statistic to examine whether a significant difference in survival existed between the genotype groups. Furthermore, we assessed the statistical association between these genotypes and survival outcomes in smoking-related head and neck cancer patients. We used a Cox proportional hazards model to conduct this analysis, which integrated age, sex, race and ethnicity, smoking status, alcohol use status, disease stage, comorbid conditions, and treatment as covariates. We additionally conducted a multivariable analysis to assess the influence of functional genetic variants on survival by employing stratification according to smoking status and treatment. Finally, we conducted a multivariable analysis to examine the combined impact of *TGF-β1* rs1800470 and *TGF-βR1* rs334348 on survival, stratifying patients according to smoking status and treatment.

## Results

### Patient characteristics

Table 1 shows the demographic and clinical characteristics of the 1403 study patients. The median follow-up duration was 37.0 months (range 0.7 to 216.0). The mean age at diagnosis was 59.7 years (range 26 to 94). Of the 1403 patients, 960 (68.4%) were men, and 443 (31.6%) were women. A total of 1159 patients (82.6%) identified as non-Hispanic White, and 244 (17.4%), as another race and ethnicity. Among the patients, 1054 (75.1%) were ever smokers, and 1021 (72.8%) were ever alcohol drinkers. More than half of the patients (812; 57.9%) presented with stage III or IV disease. The primary treatment modality was chemoradiation for 908 patients (64.7%) and surgery for 495 (35.3%). A total of 615 patients died during follow-up, 259 from smoking-related head and neck cancer, and 359 patients had disease recurrence.

**Table 1:** Characteristics of patients with smoking-related head and neck cancer (*N* = 1403)

Characteristic	No. (%) of patients
Age at diagnosis	
≤ 57 years	577 (41.1)
> 57 years	826 (58.9)
Sex	
Male	960 (68.4)
Female	443 (31.6)
Race and ethnicity	
Non-Hispanic White	1159 (82.6)
Other race and ethnicity	244 (17.4)
Smoking	
Never	349 (24.9)
Ever	1054 (75.1)
Alcohol use	
Never	382 (27.2)
Ever	1021 (72.8)
Index cancer stage	
I or II	591 (42.1)
III or IV	812 (57.9)
Comorbid conditions	
None or mild	1176 (83.8)
Moderate to severe	227 (16.2)
Treatment	
Surgery only	495 (35.3)
Others^a^	908 (64.7)
Death from any cause	
Yes	615 (43.9)
No	786 (56.1)
Death from disease	
Yes	259 (18.5)
No	1142 (81.5)
Recurrence	
Yes	359 (25.6)
No	1044 (74.4)

^a^Others: surgery and radiotherapy; surgery, radiotherapy, and chemotherapy; radiotherapy alone; and radiotherapy and chemotherapy; respectively

### Association of TGF-β1 rs1800470 and TGF-βR1 rs334348 with survival

**Fig. 1:**
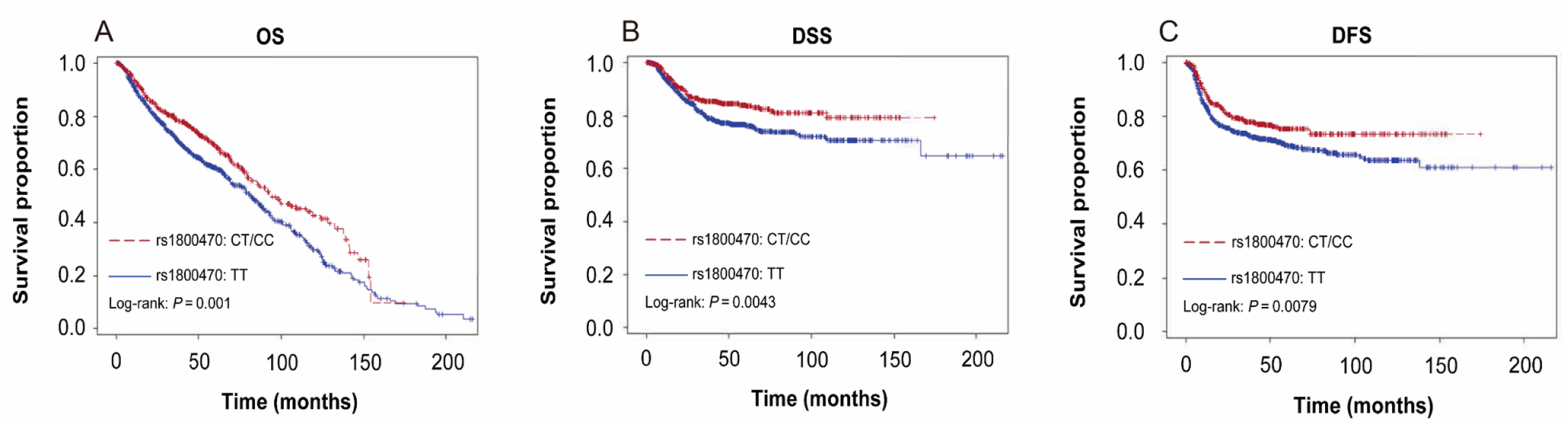
The OS, DSS, and DFS in all patients with smoking-related head and neck cancer (*N* = 1403) by (**A**) OS estimated for *TGF-β1* rs1800470 genotype, (**B**) DSS estimated for *TGF-β1* rs1800470 genotype, and (**C**) DFS estimated for *TGF-β1* rs1800470 genotype

We observed that patients with CT or CC genotype of *TGF-β1* rs1800470 experienced improved OS, DSS, and DFS compared to those with TT genotype (log-rank *P* = 0.001, *P* = 0.0043, *P* = 0.0079, respectively, Fig. 1). Furthermore, patients with CT or CC genotype had 30–35% reduced risks of death and recurrence compared to patients with TT genotype (adjusted hazard ratio [aHR], 0.70; 95% CI, 0.59 to 0.84 for OS; aHR, 0.66; 95% CI, 0.50 to 0.87 for DSS; and aHR, 0.71; 95% CI, 0.56 to 0.89 for DFS) (Table 2).

**Table 2:** Association of *TGF-β1* and *TGF-βR1* Variants with OS, DSS, and DFS of smoking-related head and neck cancer patients (*N* = 1403)

Genotypes	OS		DSS		DFS	
	Overall death/Total	aHR^a^ (95% CI); *P* value	Death, owing to disease/Total	aHR^a^ (95% CI); *P* value	Recurrence/Total	aHR^a^ (95% CI); *P* value
*TGF-β1* rs1800470						
TT^b^	432/888	1.0	184/888	1.0	248/888	1.0
CT or CC	183/515	0.70 (0.59–0.84); 0.001	75/515	0.66 (0.50–0.87); 0.0001	111/515	0.71 (0.56–0.89); 0.003
*TGF-βR1* rs334348						
AA^b^	545/1088	1.0	228/1088	1.0	307/1088	1.0
GA or GG	70/315	0.42 (0.33–0.55); < 0.0001	31/315	0.42 (0.29–0.61); < 0.0001	52/315	0.49 (0.37–0.67); < 0.0001
Combined genotypes						
TT + AA^b^	394/744	1.0	163/744	1.0	215/744	1.0
CT/CC + AA or GA/GG + TT	189/488	0.74 (0.62–0.89); 0.001	86/488	0.74 (0.57–0.97); 0.034	125/488	0.80 (0.64-1.00); 0.049
CT/CC + GA/GG	32/171	0.30 (0.21–0.44); < 0.0001	10/171	0.22 (0.12–0.43); < 0.0001	19/171	0.30 (0.19–0.49); < 0.0001

^a^Adjusted for age, sex, race and ethnicity, smoking status, alcohol use status, stage, comorbid conditions, and treatment

^b^Reference group

**Fig. 2:**
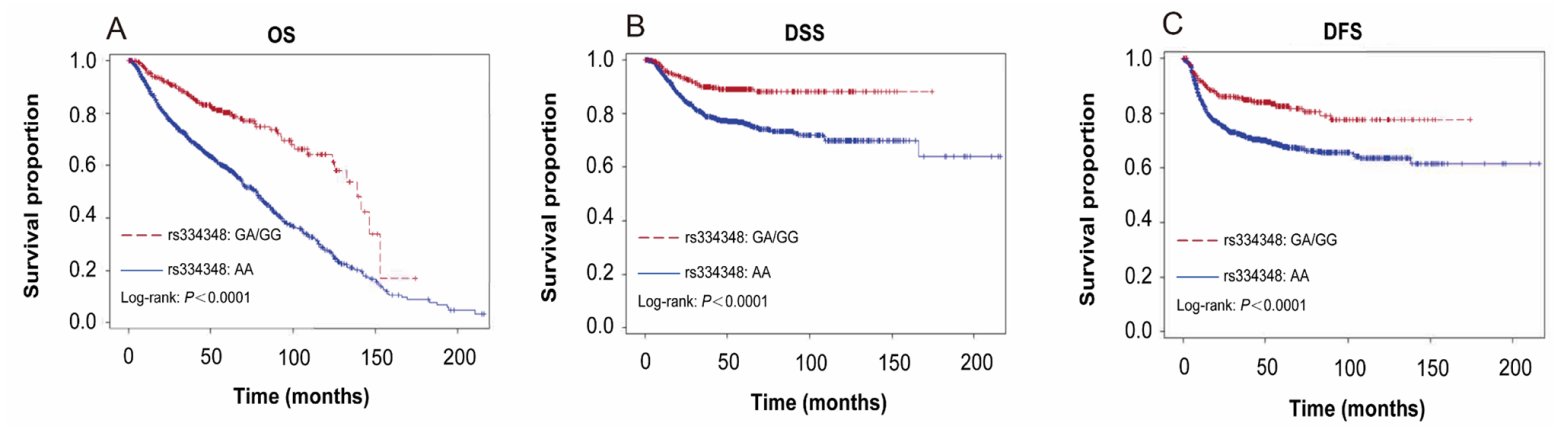
The OS, DSS, and DFS in all patients with smoking-related head and neck cancer (*N* = 1403) by (**A**) OS estimated for *TGF-βR1* rs334348 genotype, (**B**) DSS estimated for *TGF-βR1* rs334348 genotype, and (**C**) DFS estimated for *TGF-βR1* rs334348 genotype

**Fig. 3:**
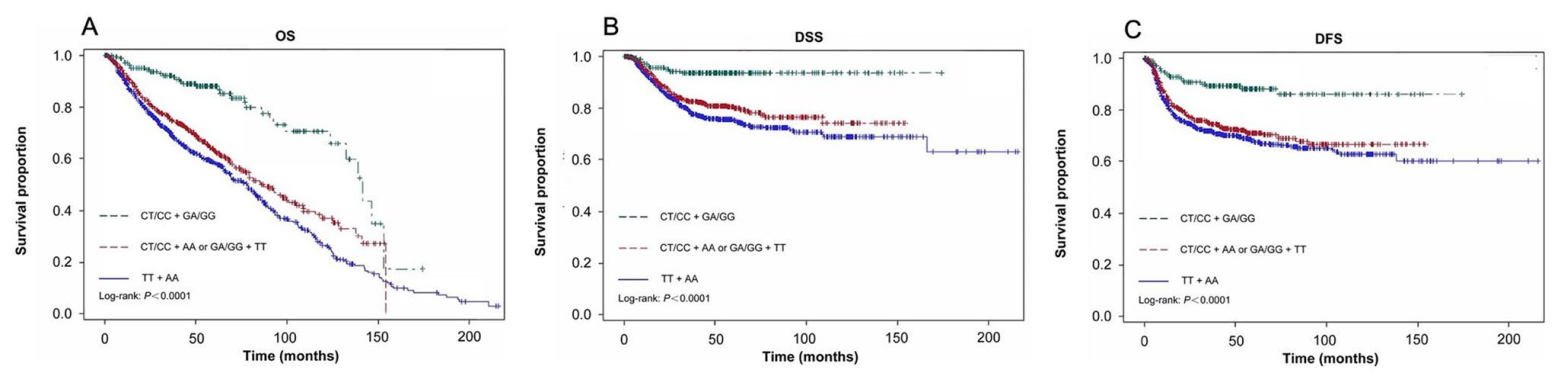
The OS, DSS, and DFS by the combined genotypes of the 2 polymorphisms in all patients with smoking-related head and neck cancer (*N* = 1403). (**A**): OS; (**B**): DSS; and (**C**): DFS, respectively

Patients with GA or GG genotype of *TGF-βR1* rs334348 experienced improved OS, DSS, and DFS compared to those with AA genotype (all log-rank *P* < 0.0001, Fig. 2). In addition, patients with GA or GG genotype had 50–60% reduced risks of death and recurrence compared to patients with AA genotype (aHR, 0.42; 95% CI, 0.33 to 0.55 for OS; aHR, 0.42; 95% CI, 0.29 to 0.61 for DSS; and aHR, 0.49; 95% CI, 0.37 to 0.67 for DFS) (Table 2). When we combined variant genotypes of the 2 SNPs, we found that the patients with combined CT/CC and GA/GG genotypes and significantly much better survival outcomes (all log-rank, *p* < 0.0001; Fig. 3) and more reduced risk for the clinical outcomes compared with those with TT and AA genotypes of the 2 SNPs (aHR, 0.30 and 95% CI, 0.21-0.044 for OS; aHR, 0.22 and 95% CI, 0.12-0.043 for DSS; and aHR, 0.30 and 95% CI, 0.19–0.49 for DFS; respectively, Table 2).

### Association of TGF-β1 rs1800470 and TGF-βR1 rs334348 with survival by smoking status

In the subset of ever smokers, patients with CT or CC genotype of *TGF-β1* rs1800470 experienced improved OS, DSS, and DFS compared to those with TT genotype. Additionally, patients with GA or GG genotype of *TGF-βR1* rs334348 experienced improved OS, DSS, and DFS compared to patients with AA genotype. Conversely, among never smokers, no significant differences by genotype were discerned in OS, DSS, or DFS (All log-rank: *P* = 0.354 for OS; *P* = 0.495 for DSS; and *P* = 0.809 for DFS for *TGF-β1* rs1800470 and *P* = 0.171 for OS; *P* = 0.987 for DSS; and *P* = 0.790 for DFS for *TGF-βR1* rs334348, respectively).

Among ever smokers, patients with CT or CC genotype of *TGF-β1* rs1800470 had 30–35% reduced risks of death and recurrence, and patients with GA or GG genotype of *TGF-βR1* rs334348 had 60–70% reduced risks of death and recurrence (aHR, 0.71; 95% CI, 0.58 to 0.87 for OS; aHR, 0.65; 95% CI, 0.48 to 0.88 for DSS; and aHR, 0.69; 95% CI, 0.53 to 0.89 for DFS for *TGF-β1* rs1800470; aHR, 0.39; 95% CI, 0.30 to 0.52 for OS; aHR, 0.33; 95% CI, 0.21 to 0.53 for DSS; and aHR, 0.43; 95% CI, 0.30 to 0.62 for DFS for *TGF-βR1* rs334348) (Table 3). In contrast, among never smokers, no significant associations were found between the genotypes of *TGF-β1* rs1800470 and *TGF-βR1* rs334348 and survival outcomes (Table 3).

**Table 3:** Association of *TGF-β1* and *TGF-βR1* variants with OS, DSS, and DFS of smoking-related head and neck cancer patients stratified by smoking status

Genotypes	OS		DSS		DFS	
	Overall death/Total	aHR^a^ (95% CI); *P* value	Death, owing to disease/Total	aHR^a^ (95% CI); *P* value	Recurrence/Total	aHR^a^ (95% CI); *P* value
Ever smoker patients (*N* = 1054)						
*TGF-β1* rs1800470						
TT^b^	349/670	1.0	155/670	1.0	203/670	1.0
CT or CC	155/384	0.71 (0.58–0.87); 0.0008	61/384	0.65 (0.48–0.88); 0.0059	86/384	0.69 (0.53–0.89); 0.0049
*TGF-βR1* rs334348						
AA^b^	449/828	1.0	196/828	1.0	254/828	1.0
GA or GG	55/226	0.39 (0.30–0.52); < 0.0001	20/226	0.33 (0.21–0.53); < 0.0001	35/226	0.43 (0.30–0.62); < 0.0001
Never smoker patients (*N* = 349)						
*TGF-β1* rs1800470						
TT^b^	83/218	1.0	29/218	1.0	45/218	1.0
CT or CC	28/131	0.70 (0.44–1.10); 0.122	14/131	0.70 (0.36–1.39); 0.309	25/131	0.82 (0.49–1.36); 0.435
*TGF-βR1* rs334348						
AA^b^	96/260	1.0	32/260	1.0	53/260	1.0
GA or GG	15/89	0.59 (0.34–1.04); 0.102	11/89	0.77 (0.37–1.57); 0.466	17/89	0.71 (0.40–1.25); 0.234

^a^Adjusted for age, sex, race and ethnicity, alcohol use status, stage, comorbid conditions, and treatment

^b^Reference group

### Association of TGF-β1 rs1800470 and TGF-βR1 rs334348 with survival by treatment

Among the patients treated with chemoradiation, patients with CT or CC genotype of *TGF-β1* rs1800470 experienced improved OS, DSS, and DFS compared to those with TT genotype, and patients with GA or GG genotype of *TGF-βR1* rs334348 experienced improved OS, DSS, and DFS compared to those with AA genotype. Conversely, among patients treated with surgery only, no significant genotype-related differences were discerned in OS, DSS, or DFS (All log-rank: *P* = 0.110 for OS; *P* = 0.121 for DSS; and *P* = 0.325 for DFS for *TGF-β1* rs1800470 and *P* = 0.171 for OS; *P* = 0.174 for DSS; and *P* = 0.270 for DFS for *TGF-βR1* rs334348, respectively).

Among patients treated with chemoradiation, patients with CT or CC genotype of *TGF-β1* rs1800470 had 30% reduced risks of death and recurrence, and patients with GA or GG genotype of *TGF-βR1* rs334348 had 50–60% reduced risks of death and recurrence (aHR, 0.71; 95% CI, 0.57 to 0.87 for OS; aHR, 0.70; 95% CI, 0.52 to 0.94 for DSS; and aHR, 0.70; 95% CI, 0.54 to 0.92 for DFS for *TGF-β1* rs1800470; aHR, 0.40; 95% CI, 0.30 to 0.54 for OS; aHR, 0.39; 95% CI, 0.26 to 0.60 for DSS; and aHR, 0.44; 95% CI, 0.30 to 0.63 for DFS for *TGF-βR1* rs334348) (Table 4). In contrast, among patients treated with surgery only, no significant associations were found between the genotypes of *TGF-β1* rs1800470 and *TGF-βR1* rs334348 and survival outcomes (Table 4).

**Table 4:** Association of *TGF-β1* and *TGF-βR1* variants with OS, DSS, and DFS of smoking-related head and neck cancer patients stratified by treatment

Genotypes	OS		DSS		DFS	
	Overall death/Total	aHR^a^ (95% CI); *P* value	Death, owing to disease/Total	aHR^a^ (95% CI); *P* value	Recurrence/Total	aHR^a^ (95% CI); *P* value
Patients treated with surgery only (*N* = 495)						
*TGF-β1* rs1800470						
TT^b^	97/297	1.0	27/297	1.0	53/297	1.0
CT or CC	45/198	0.75 (0.52–1.09); 0.131	10/198	0.51 (0.24–1.07); 0.104	28/198	0.71 (0.45–1.14); 0.153
*TGF-βR1* rs334348						
AA^b^	124/372	1.0	31/372	1.0	64/372	1.0
GA or GG	18/123	0.50 (0.30-1.00); 0.056	6/123	0.56 (0.23–1.37); 0.206	17/123	0.68 (0.40–1.17); 0.166
Patients treated with chemoradiation (*N* = 908)						
*TGF-β1* rs1800470						
TT^b^	335/591	1.0	157/591	1.0	195/591	1.0
CT or CC	138/317	0.71 (0.57–0.87); 0.0011	65/317	0.70 (0.52–0.94); 0.018	83/317	0.70 (0.54–0.92); 0.009
*TGF-βR1* rs334348						
AA^b^	421/716	1.0	197/716	1.0	243/716	1.0
GA or GG	52/192	0.40 (0.30–0.54); < 0.0001	25/192	0.39 (0.26–0.60); < 0.0001	35/192	0.44 (0.30–0.63); < 0.0001

^a^Adjusted for age, sex, race and ethnicity, smoking status, alcohol use status, stage, and comorbid conditions

^b^Reference group

### Validation for associations between TGF-β1 rs1800470 and TGF-βR1 rs334348 and survival outcomes using the TCGA dataset

**Fig. 4:**
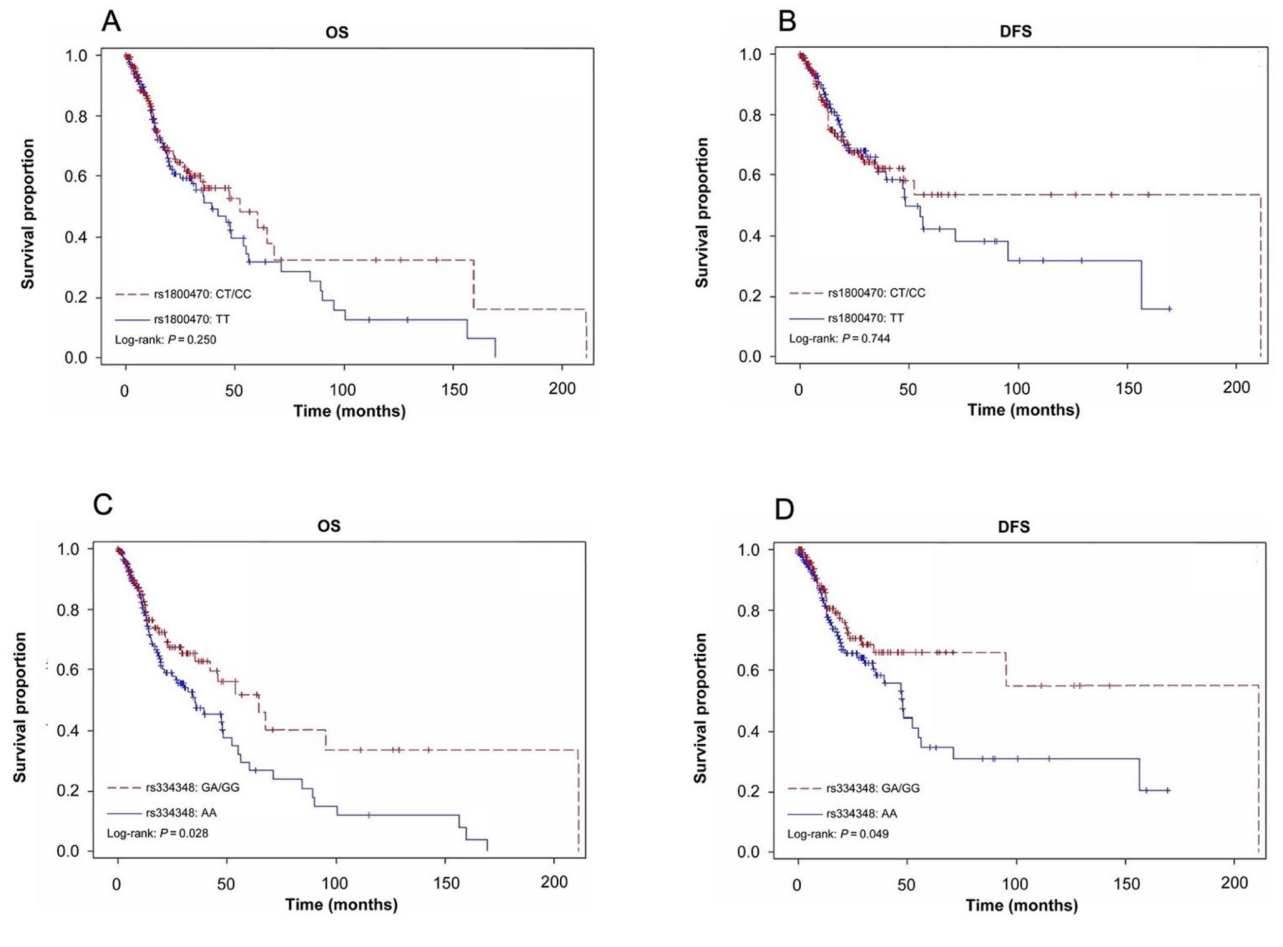
The OS, DSS, and DFS by *TGF-β1* rs1800470 and *TGF-βR1* rs334348 genotypes in the patients with smoking-related head and neck cancer from TCGA (*N* = 312). (A) OS estimated for *TGF-β1* rs1800470 genotype, (B) DFS estimated for *TGF-β1* rs1800470 genotype, (C) OS estimated for *TGF-βR1* rs334348 genotype, and (D) DFS estimated for *TGF-βR1* rs334348 genotype

For validation the findings mentioned above, a total of 312 smoking-related head and neck cancer patients with epidemiological, clinical, and survival data from the TCGA dataset as well as the genotyping data of the 2 SNPs from the NIH dbGaP were available for validation analysis (Table 5). As shown in Fig. 4, the similar survival differences from the TCGA patients were observed for the two SNPs, in particular, the differences of OS and DFS were statistically significant for *TGF-βR1* rs334348 (Log-rank, *p* = 0.028 for OS and *p* = 0.049 for DFS, Fig. 4). Moreover, after adjustment with several prognostic confounders available in the TCGA dataset, the similar association patterns were observed among the patient in the TCGA for the 2 SNPs, especially the significant associations between the variant genotypes and risk of overall deaths and disease recurrence were found for ***TGF-****βR1* rs334348 (aHR, 0.64 and 95% CI, 0.43–0.95 for OS and aHR, 0.63 and 95% CI, 0.40–0.98 for DFS, Table 6). The similar associations were found for ***TGF-****βR1* rs334348 when the patients were stratified by patients’ smoking status (Table 7).

**Table 5:** Characteristics of patients with smoking-related head and neck cancers from the TCGA (*N* = 312)

Characteristic	No. (%) of patients
Age at diagnosis	
≤ 57 years	107 (34.3)
> 57 years	205 (65.7)
Sex	
Male	216 (69.2)
Female	96 (30.8)
Race and ethnicity	
Non-Hispanic White	312 (100)
Smoking	
Never	62 (19.9)
Ever	241 (77.2)
Unknown	9(2.9)
Alcohol use	
Never	102(32.7)
Ever	204 (65.4)
Unknown	6(1.9)
Index cancer stage	
I or II	72 (23.1)
III or IV	240 (76.9)
Death from any cause	
Yes	112 (35.9)
No	200 (64.1)
Recurrence	
Yes	83 (26.6)
No	229 (73.4)

**Table 6:** Association of *TGF-β1* and *TGF-βR1* Variants with OS and DFS of smoking-related head and neck cancer patients From the TCGA (*N* = 312)

Genotypes	OS		DFS	
	Overall death/Total	aHR^a^ (95% CI); *P* value	Recurrence/Total	aHR^a^ (95% CI); *P* value
*TGF-β1* rs1800470				
TT^b^	60/141	1.0	41/141	1.0
CT or CC	52/171	0.72 (0.55–1.17); 0.252	42/171	0.78 (0.60–1.44); 0.745
*TGF-βR1* rs334348				
AA^b^	74/184	1.0	55/184	1.0
GA or GG	38/128	0.64 (0.43–0.95); 0.029	28/128	0.63 (0.40–0.98); 0.048

^a^Adjusted for age, sex, smoking status, alcohol use status, and stage

^b^Reference group

**Table 7:** Association of *TGF-β1* and *TGF-βR1* variants with OS and DFS of smoking-related head and neck cancer patients from the TCGA, stratified by smoking status^c^ (*N* = 312)

Genotypes	OS		DFS	
	Overall death/Total	aHR^a^ (95% CI); *P* value	Recurrence/Total	aHR^a^ (95% CI) ; *P* value
Never smoker patients (*N* = 62)				
*TGF-β1* rs1800470				
TT^b^	12/32	1.0	8/32	1.0
CT or CC	7/30	0.64 (0.25–1.66); 0.366	9/30	0.73 (0.50–3.35); 0.598
*TGF-βR1* rs334348				
AA^b^	15/39	1.0	12/39	1.0
GA or GG	4/23	0.42(0.14–1.28); 0.127	5/23	0.67 (0.23–1.93); 0.461
Ever smoker patients (*N* = 241)				
*TGF-β1* rs1800470				
TT^b^	46/105	1.0	33/105	1.0
CT or CC	41/136	0.78 (0.53–1.25); 0.349	31/136	0.83 (0.51–1.36); 0.461
*TGF-βR1* rs334348				
AA^b^	57/142	1.0	43/142	1.0
GA or GG	30/99	0.63 (0.44-1.00); 0.049	21/99	0.63 (0.37–0.99); 0.048

^a^Adjusted for age, sex, alcohol use status, and stage

^b^Reference group

^c^Missing: No information on smoking status for 9 cases

## Discussion

This study explored potential associations between the *TGF-β1* rs1800470 and *TGF-βR1* rs334348 genetic variants and the clinical outcomes of patients diagnosed with smoking-related head and neck cancer within a cohort of 1403 patients. Our findings suggest that these genetic variants are significantly associated with the risk of death and disease recurrence. Notably, the impact of the *TGF-β1* rs1800470 and *TGF-βR1* rs334348 genetic variants on clinical outcomes was particularly pronounced in the subgroups of ever smokers and patients treated with chemoradiation.

miRNAs, functioning as gene regulators, potentially contribute to cancer progression, increased susceptibility to cancer, and the prognosis of patients with cancer [27]. This involvement might be orchestrated through miRNA-mediated regulation of gene expression, which could be modulated by functional genetic variants residing within the miRNA binding sites; in particular, SNP within the miRNA binding sites of the 3’ untranslated region may play a role in the complex mechanisms underlying oncogenesis [17]. Previous studies demonstrated that *TGF-β1* rs1800470 and *TGF-βR1* rs334348 genetic variants located in the miRNA binding sites were critically associated with the risk of cancer development and clinical outcome for various cancers [14, 28]. Thus, comprehensive exploration of the associations between these genetic variants and clinical outcomes may pave the way for the establishment of more appropriate and precise personalized treatments.

Associations between the prognosis of smoking-related head and neck cancer and *TGF-β1* rs1800470 and *TGF-βR1* rs334348 were identified in several previous studies. Lundberg et al. [29] observed better OS and DFS in chemoradiotherapy-treated HNSCC patients carrying the C-allele of *TGF-β1* rs1800470 than in patients carrying the TT genotype. Abakay et al. [30] focusing on primary laryngeal SCC within the Turkish population, found a noteworthy association between *TGF-β1* polymorphisms and cancer susceptibility. In the patient cohort, a notable augmentation in the prevalence of the GC genotype and C allele associated with *TGF-β1* rs1800471 was observed, indicating a significant association with susceptibility to laryngeal SCC. Conversely, no such association was evident for the *TGF-β1* rs1800470. Our results suggest a protective role of the rs1800470 CT/CC in smoking-related head and neck cancer prognosis, the difference in these findings could be attributed to several factors including the ethnic composition of the study populations, the genetic background, the environmental exposures, and the specific characteristics of the patient cohorts, such as the tumor stage and treatment modalities. Moreover, these might be through mechanisms that involve a more effective immune response or a different pattern of cytokine expression. The functional genetic variants of *TGF-β1* were verified via in vitro experiments. In a case-control study by Hu et al. [21]. , it was found that patients carrying the *TGF-β1* rs1800469 -509T allele had a significantly reduced risk of developing nasopharyngeal carcinoma compared to those who did not carry this allele. Moreover, the mRNA expression level of -509CC and − 509CT was remarkably higher than that of -509TT in nasopharyngeal carcinoma tissues. Even though Hu et al. [21] found that the association between *TGF-β1* rs1800470 and the survival of patients with nasopharyngeal carcinoma was not significant, they further elucidated that *TGF-β1* functional genetic variants may affect malignancy susceptibility. Knobloch et al. [31] identified a significant association between *TGF-βR1* rs334348 and both the occurrence and advancement of HNSCC. Chen et al. [32] discovered that the *TGF-βR1* rs334348 polymorphism may modulate the progression of oral cancer by regulating the expression of TGF-βR1 and diminishing its ability to phosphorylate Smad-2 and Smad-3. Moreover, the researchers detected a synergistic association between *TGF-βR1* rs334348 GA or GG genotype and the improved DSS of patients treated with radiotherapy, implying the potential clinical relevance of this genetic alteration in cancer management. In our present study, all patients with *TGF-β1* rs1800470 CT or CC genotype and *TGF-βR1* rs334348 GA or GG genotype had decreased risks of death and recurrence, and these associations were especially pronounced in the subgroups of ever smokers and patients treated with chemoradiation. These previous studies suggest a strong association between *TGF-β1* and *TGF-βR1* functional genetic variants and the prognosis of patients with smoking-related head and neck cancer, which supports our results and indicates the potential of *TGF-β1* and *TGF-βR1* to be a key factor in predicting patient’s treatment responsiveness and the prognosis of our studied patients.

Our previous findings [15, 18] demonstrated a notable association between *TGF-β1* rs1800470 and survival in patients with HPV16-positive oropharyngeal cancer following definitive radiotherapy. These findings were consistent with our present study and imply that this genetic variant could induce a functional alteration and potentially influence treatment responses, impacting clinical results. However, the results of other studies across diverse cancer types may not be consistent with our current study’s findings concerning the impacts of *TGF-β1* rs1800470 and *TGF-βR1* rs334348. Both *TGF-β1* rs1800470 and *TGF-βR1* rs334348 act as oncogenes in gastric cancer. Juarez et al. [19] demonstrated that patients with gastric adenocarcinoma carrying the *TGF-β1* rs1800470 TT or CT genotype had a decreased survival rate compared to those with the CC genotype. Similarly, He et al. [33] found a significant association between the *TGF-βR1* rs334348 GG genotype and increased risk for gastric cancer. The differing prognostic relevance of *TGF-β1* rs1800470 and *TGF-βR1* rs334348 across an array of cancer types could be attributed to an assortment of factors, including the simultaneous existence of other oncogenic agents (e.g., HPV), the heterogeneity of tumors, differing molecular subclasses, and unique alterations within the tumor microenvironment.

Our present study suggests that *TGF-β1* rs1800470 and *TGF-βR1* rs334348 genetic variants may act as a protective factor in ever smokers with smoking-related head and neck cancer. The observed protective effect may be linked to the modulation of pro-inflammatory and immunosuppressive responses caused by smoking-induced expression and secretion of pro-inflammatory factors [10, 34, 35]. This process may alter responsiveness to treatment, thereby reducing the risk of mortality and disease recurrence. Intriguingly, when we stratified our cohort by smoking status and treatment, we found that the associations between *TGF-β1* rs1800470 CT or CC and *TGF-βR1* rs334348 GA or GG and prognosis were similar in ever smokers and patients treated with chemoradiation. This may be due to the DNA damage inflicted on cancer cells by radiotherapy and chemotherapy. Smoking-related head and neck cancer patients may also experience somatic genetic alterations, and these hypothetical functional genetic variants may enable a significant number of tumor cells to evade the immune system and apoptotic responses, thus resulting in variable sensitivity to chemoradiation. However, further studies are required to confirm these hypotheses.

The roles of *TGF-β1* rs1800470 and *TGF-βR1* rs334348 in cancer could potentially be explained by the fact that these polymorphisms are located in miRNA binding sites. Nicoloso et al. [14] explored the differences in function for *TGF-β1* rs1800470 and *TGF-βR1* rs334348 and found that the *TGF-β1* rs1800470 polymorphism is situated at the miRNA187 binding site, with the T to C transition within this variant affecting the minimum free energy in the miRNA187::TGF-β1-mRNA complex. Moreover, Nicoloso et al. [14] performed the miR-187 luciferase assay and found that *TGF-β1* rs1800470 affected protein levels of TGF-β1 by its genotypes, respectively. In addition, they evaluated the effect of *TGF-βR1* rs334348 on miR-628-5p regulation of TGF-βR1. By overexpressing miR-628-5p in different cell lines, they observed the different effects of the genotypes of this single nucleotide polymorphism on the activity of TGF-βR1 protein levels in a cell-specific manner. This change in binding dynamics may influence miRNA gene regulation and the expression and functionality of TGF-β1 and TGF-βR1, potentially impacting both cancer susceptibility and prognosis.

The significantly greater effect of *TGF-βR1* rs334348 than of *TGF-β1* rs1800470 on reducing the risk of death and recurrence in patients with smoking-related head and neck cancer in the present study may be attributed to the location of *TGF-β1* rs1800470 outside the 3’ untranslated region of *TGF-β1*, which might constrain its role in miRNA binding activity. We hypothesize that *TGF-β1* rs1800470 could exert its influence through various mechanisms. From a biological standpoint, while the exact association of *TGF-β1* rs1800470 with miRNA function remains an enigma, it is possible that *TGF-β1* rs1800470 influences the expression level of TGF-β1 and the activity of the TGF-β1 signaling pathway. Such alterations could have downstream effects on biological processes, including cell proliferation, apoptosis, and inflammation [36]. The impact of these two single nucleotide polymorphisms on survival might function through an array of mechanisms, indicating the potential involvement of additional mediatory elements apart from the expression of the two respective genes [37]. The precise molecular mechanisms through which *TGF-β1* rs1800470 and *TGF-βR1* rs334348 impact the incidence and prognosis of smoking-related head and neck cancer require further exploration in both in vitro and in vivo experimental models.

To our knowledge, this is the first thorough research that further elucidates the associations between *TGF-β1* rs1800470 and *TGF-βR1* rs334348 and clinical outcomes in patients with smoking-related head and neck cancer overall and stratified by smoking status and treatment. These functional genetic variants markedly contribute to the reduction of death and disease recurrence risk among smoking-related head and neck cancer patients, particularly in ever smokers and patients treated with chemoradiation. Our findings suggest that utilizing pathway-based strategies may facilitate a more precise risk assessment in the future, leading to a deeper understanding of the etiology and prognosis of smoking-related head and neck cancer. Utilizing personalized treatment by considering each patient’s unique genetic makeup, enables a more targeted and effective intervention to combat the disease.

Our study has some limitations that should be addressed. First, as most of the patients were non-Hispanic White, the generalizability of our findings to other racial and ethnic populations may be limited, for which a larger study is needed for validation in other different genetic background. Second, the interpretation of some key findings in the stratified analysis may be constrained by the relatively small sample size within each subgroup. Third, given that the overarching study was both hospital-based and retrospective, potential selection and confounding biases may have impacted the patient cohort studied. Finally, considering the constraints of our limited sample size and fewer instances of outcome events in certain subsets, it is plausible that our findings deemed statistically significant may be attributable to random fluctuation. To bolster our conclusions, it would be beneficial to conduct additional studies that involve more participants.

Above all, our current research suggests that the *TGF-β1* rs1800470 and *TGF-βR1* rs334348 functional genetic variants may alter survival outcomes in smoking-related head and neck cancer patients, especially in the subgroups of ever smokers and patients treated with chemoradiation. These genetic variants may serve as prognostic predictors for patients with smoking-related head and neck cancer. They may play a role in advancing the field of personalized cancer therapy, thereby improving patient survival rates and quality of life. Nevertheless, for a thorough validation of these associations and the use of these variants as clinical prognostic biomarkers, additional research is needed to affirm our findings and further elucidate the molecular mechanisms underlying these observed associations.

## Data Availability

The data generated in this study are not publicly available due to information that could compromise patient privacy or consent but are available upon reasonable request from the corresponding author. The genotyping data from the genome-wide association study of SCC of head and neck from MD Anderson Cancer Center have been deposited in dbGaP (accession #: phs001173.v1.p1).

## Abbreviations

CI: Confidence interval
DFS: Disease-free survival
DSS: Disease-specific survival
HNSCC: Head and neck squamous cell carcinoma
HPV: Human papillomavirus
HR: Hazard ratio
miRNA: microRNA
OR: odds ratio
OS: Overall survival
SNP: Single nucleotide polymorphism
TGF-β1: Transforming growth factor β1
TGF-βR1: Transforming growth factor β receptor 1
